# Nitrate and Nitrite in the Diet: Protective and Harmful Effects in Health and Disease

**DOI:** 10.1007/s13668-025-00678-5

**Published:** 2025-06-30

**Authors:** Aneta Sokal-Dembowska, Sara Jarmakiewicz-Czaja, Jacek Tabarkiewicz, Rafał Filip

**Affiliations:** 1 Department of Dietetics, Faculty of Health Sciences and Psychology, Medical College of Rzeszow University, University of Rzeszow, Rzeszow, 35- 959 Poland; 2 Department of Human Immunology, Institute of Medical Sciences, Medical College of Rzeszow University, University of Rzeszow, Rzeszow, 35-959 Poland; 3 Laboratory for Translational Research in Medicine, Centre for Innovative Research in Medical and Natural Sciences, Medical College of Rzeszow University, University of Rzeszow, Rzeszow, 35-959 Poland; 4 Gastroenterology Clinic, Center for Comprehensive Treatment of Inflammatory, Bowel Disease Regional Hospital No. 2 in Rzeszow, Rzeszow, 35-301 Poland; 5 Department of Internal Medicine, Faculty of Medicine, Medical College of Rzeszow Univeristy, University of Rzeszow, Rzeszow, 35-959 Poland

**Keywords:** Daily intake, Health risks, Nitrates, Nitrites, Thyroid disorders

## Abstract

**Purpose of Review:**

The environmental presence of excess nitrogen poses a significant threat to biodiversity, human health, and climate stability. Conversely, nitrogen deficiency can hinder our ability to sustainably provide sufficient food for the global population. The impact of nitrates and nitrites on human health is intricately tied to their concentrations in water and food sources. The aim of the following article was to summarize the data on the effects of nitrates on health, with particular emphasis on their effect on the thyroid gland.

**Recent Findings:**

Current scientific findings indicate that these compounds can have dual effects, both beneficial and harmful, on health. Dietary nitrates are naturally abundant in fruits and vegetables or can be introduced as additives, notably in processed and cured meats. Nitrate supplementation has shown promise in enhancing physical performance, influencing gut microbiota composition, and increasing short-chain fatty acid production. Studies suggest that nitrate consumption can effectively reduce inflammation and counter the deleterious effects of oxidative stress in the body. However, the addition of nitrites as preservatives may lead to the formation of nitrosamines, known carcinogens that detrimentally impact cardiovascular and metabolic functions.

**Summary:**

Excessive nitrite intake from animal sources has been linked to an increased risk of thyroid cancer, particularly among women. Nevertheless, a moderate intake of naturally occurring nitrates from food sources demonstrates potential benefits such as blood pressure regulation, improved vascular endothelial function, and enhanced tissue insulin sensitivity. These positive effects might mitigate risk factors associated with developing complications in thyroid disease and warrant further investigation.

## Introduction

According to Stein and Klotz, the fate of humanity largely depends on our ability to control the nitrogen cycle. Undoubtedly, a positive aspect of the use of nitrogen fertilizers is that its use makes it possible to produce food that keeps almost half of the world’s population alive, and this is made possible by the Haber-Bosh method (industrial fixation of N2 into ammonia, NH3) [1].

**Fig. 1:**
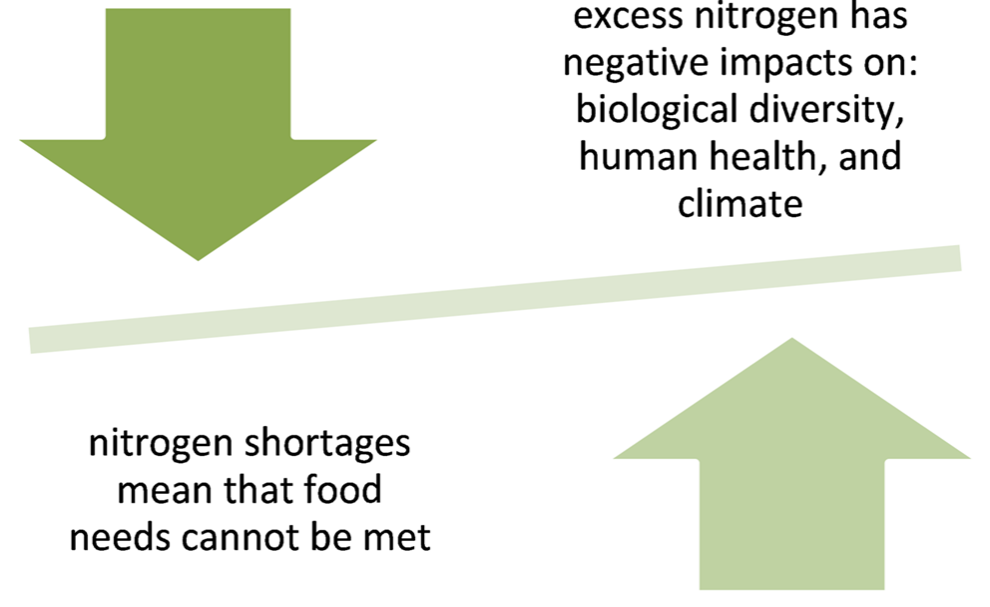
Nitrogen excess and deficiency in the environment. Based on Stein et al. 2016 [1]

Nitrates are the main source of nitrogen for terrestrial plants. Nitrogen is also a major yield-forming ingredient, and its use is increasing almost in direct proportion to the growth of the world’s population. Moreover, global grain production and fertilizer use are closely linked. A particularly rapid increase in the consumption of nitrogen fertilizers and grain production was observed in the years 1960–1990 [2]. This seems particularly significant given the ever increasing level of hunger in the world, and the Covid-19 pandemic in recent years has contributed to this increase [3]. The global population facing chronic hunger stood 735 mln in 2022, up from 613 mln in 2019 [4]. Unfortunately, the increase in the use of chemical fertilisers in previous years, in addition to the increase in agricultural crops, also had an impact on the change in the nitrogen cycle, which contributed to environmental degradation (Fig. 1) [1]. There-fore, the implementation of the Sustainable Development Goals, among which the elimination of hunger is in second place, while achieving food security and maintaining sustainable agriculture, can bring beneficial changes both to in the field of public health and environmental [5]. In Poland, a program has been introduced to reduce water pollution with nitrogen from agricultural sources based on the regulations contained in Directive 2000/60/EC (the so-called Water Framework Directive), which ap-plies to all countries belonging to the European Union [6].

Nitrates and nitrites occur naturally in water and soil; therefore, they can be pre-sent in plants. There are also nitrites used as food preservatives (sodium nitrite– E249, potassium nitrite– E250) and nitrates (sodium nitrate– E251, potassium nitrate– E252) of animal origin in the process of its processing [7].

The nitrogen ion is considered safe, however, due to the action of anaerobic bacteria, about 20% can be converted to nitrites, which have a more toxic effect [7]. Nitrites form N-nitroso compounds in the gastrointestinal tract, which are potential human carcinogens [8]. Their impact on the body largely depends on their concentration in water and food [9], which can have both negative and beneficial effects on health. The following article is a summary of the effects of nitrates on health, with particular emphasis on their effects on the thyroid gland.

## Nitrates– Definition, Prevalence, Application

Nitrogen makes up 78% of the Earth’s atmosphere and can occur in both non-reactive (N2) and reactive (Nr) forms. The reactive form includes nitrogen oxides, reduced nitrogen, nitrous oxide, nitric acid and other organic and inorganic forms. The inorganic form of nitrogen is found in the environment, mainly in the top layer of the soil, and is available to most living organisms [10]. In order for the chemically inert nitrogen to be used, it must be converted into “fixed” nitrogen [11]. The main processes of the nitrogen cycle are ammonification, nitrification, denitrification, and anammox [1]. The nitrogen that plants can use is converted into ammonia, nitrites (NO2), and nitrates (NO3). More than 90% of nitrogen is absorbed from the soil by plants. Soils can also be enriched with ammonium nitrogen, which is then converted into nitrate as a result of nitrification [11].

### Nitrogen Losses in the Environment

The greatest nitrogen losses are observed during the oxidation of ammonia, and the rate of change is influenced by factors such as soil pH, temperature and humidity. This causes the loss of NH3 into the atmosphere and acid rains, which promotes the eutrophication of some surface waters. The increase in the use of fertilizers as a source of nitrogen promotes the production of N2O, which is responsible for the depletion of the ozone layer. It may be released into the stratosphere, among others, as a result of the combustion of fossil fuels and as a result of natural processes during the production of nitrogen in the soil. Additionally, nitrates may be washed out and moved deep-er into the soil profile as a result of long-term rainfall, which contributes to the deterioration of the quality of surface and ground soils [12].

**Fig. 2:**
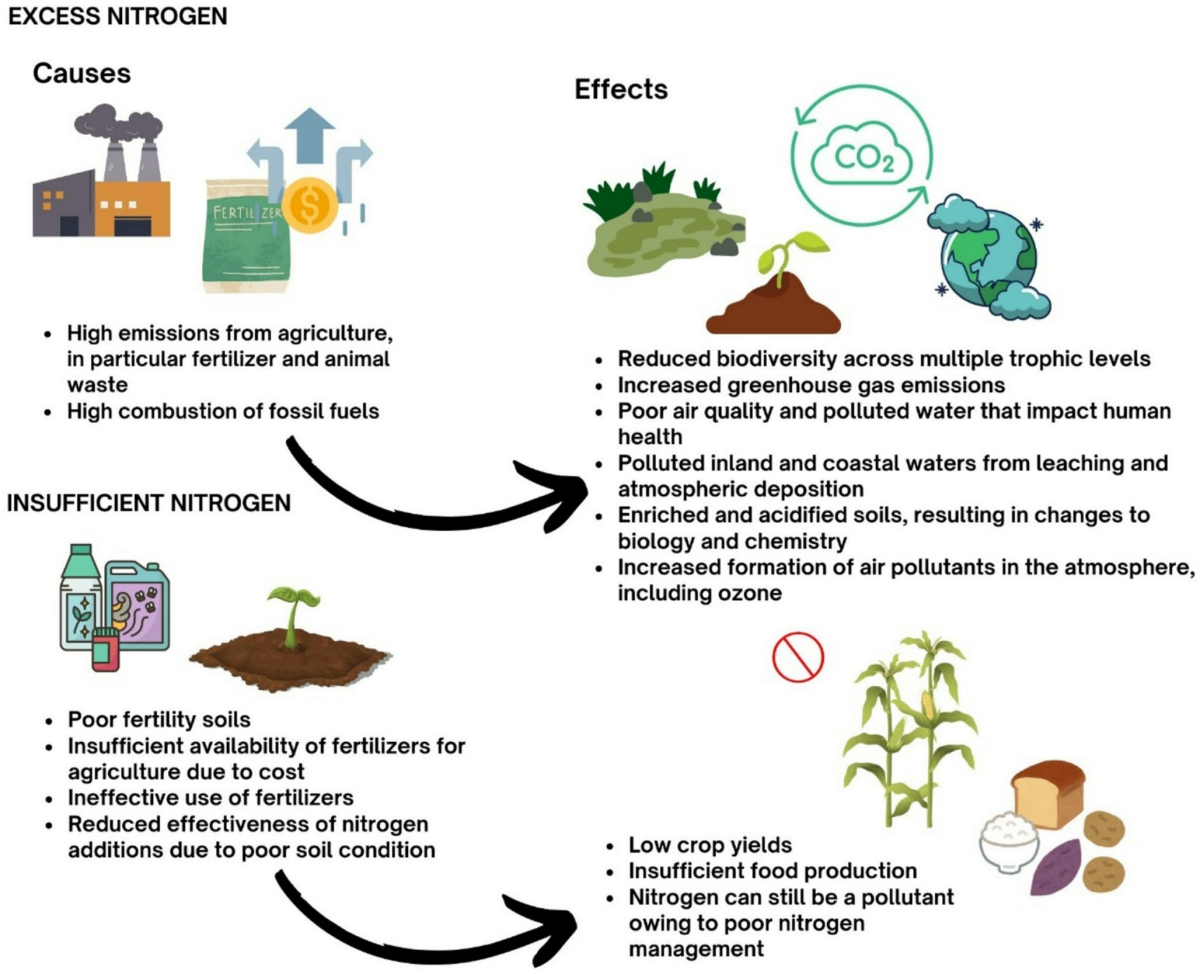
Causes and effects of excess and deficiency of nitrogen in the environment. Based on Stevens 2019 [14]

According to Dimpek et al., there is a need to improve the efficiency of fertilizer application through various types of chemical, biological and nanotechnological modifications. Fertilizers with increased efficiency can be obtained in two ways, by regulating the rate of urea hydrolysis– the addition of a synthetic or natural urease inhibitor. The second is to reduce the nitrification rate of ammonium (NH4) to ammonia [13]. Stevens points to the great need to address excess nitrogen in the environment. Nitro-gen losses can be prevented, among others, by: increased availability of fertilizers, education of farmers in order to effectively use fertilizers and soil management (Fig. 2) [14]. Total nitrogen and nitric nitrogen are among the main anthropogenic factors affecting the environmental gradient. Thus contributing to the degradation of aquatic ecosystems [15]. Groundwater pollution is a major problem in South-West Romania, among others. In these areas, strict control of water is recommended before it is al-lowed to be consumed in order to reduce health risks. In addition, MititeluIonuș et al., who conducted an analysis in the area, recommend farmers to reduce the amount of fertilizer applied over a certain period of time and to use compost. They also recommend strengthening water policy reforms in order to introduce appropriate legal regulations [16]. Nitrogen as a biogenic element is responsible for the process of eutrophication of surface waters, leading to the development of algae and cyanobacteria, thus leading to the destruction of life at the bottom of a water reservoir [17]. Due to the high content of nitrates in water in some European Union countries, the European Commission has drawn up a directive (Council Directive (91/676/EEC) of 12 December 1991 on the protection of waters against pollution caused by nitrates from agricultural sources, the so-called Nitrates Directive (Journal of Laws UE.L.1991.375.1 of 1991.12.31)) aimed at protecting human health and aquatic ecosystems. The Directive obliges Member States to apply good agricultural practice and adequate monitoring of water status [18]. In Poland, these activities are described in the Water Law Act of 20 July 2017 (Journal of Laws 2017, item 1566, as amended) [19].

Particulate matter (PM) poses a substantial challenge to both human health and environmental quality. Due to their diameters and their ability to be differentially de-posited in the respiratory tract, two classes of particles are distinguished: respirable and nonrespirable particles. According to the World Health Organization, exposure to particulate matter contributes to 4.2 million deaths worldwide each year. PM can lead to DNA mutations and the development of many chronic diseases [20]. Agriculture is one of the largest anthropogenic sources of PM in the environment. Research results indicate that the use of pig and poultry manure has a crucial impact on particle emissions. Therefore, the use of nitrogen oxide-rich manure is a major contributor to PM emissions with a high potential for particulate emissions [21].

## Exposure Pathways to Nitrates

**Fig. 3:**
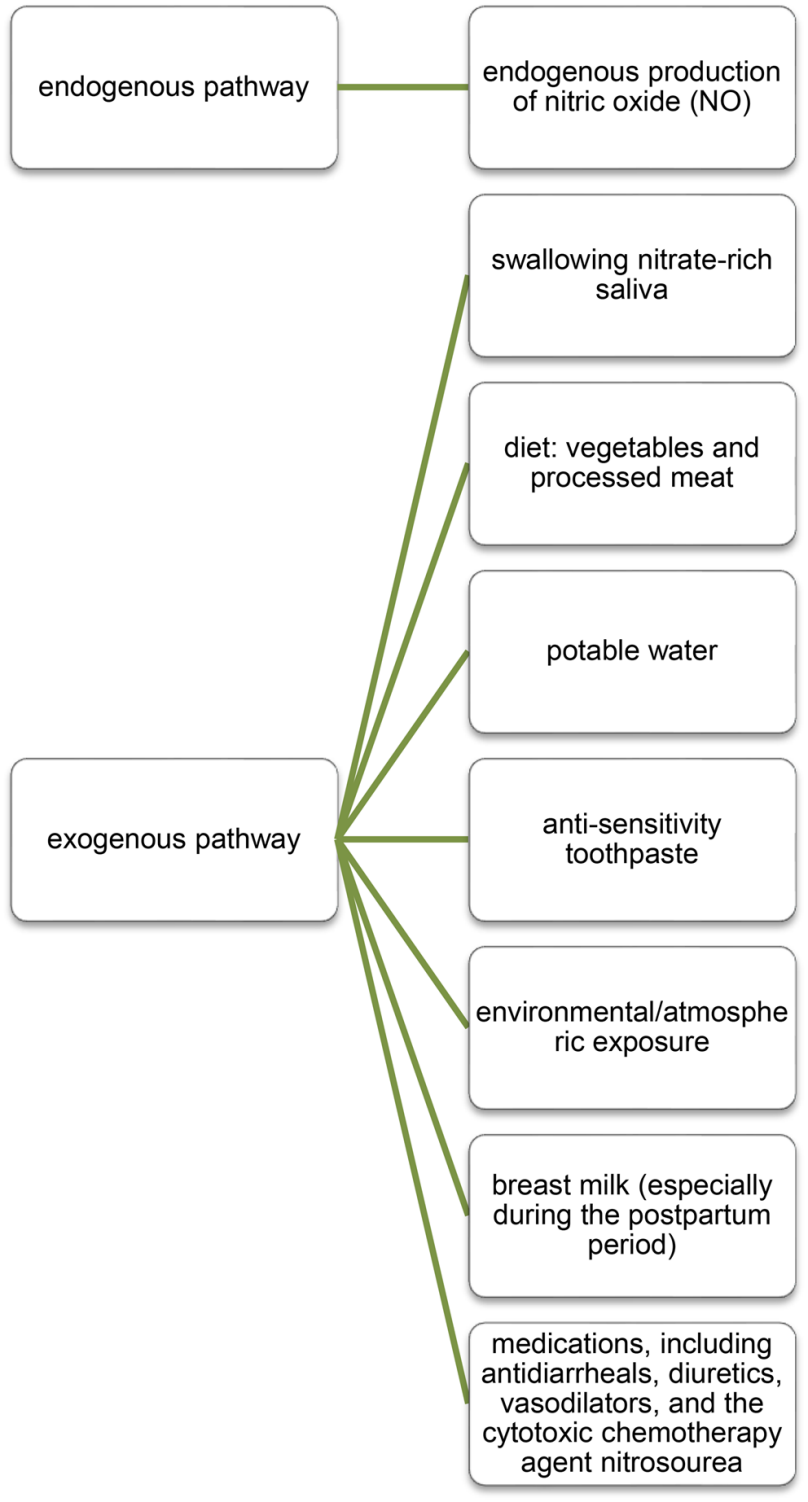
Basic exposure pathways to nitrates and nitrites. Based on Bryan et al. 2015 [22]

There are several main sources of exposure to nitrate and its metabolite nitrite. These include, among others, environmental/atmospheric pollutants, and thus the presence of compounds in food or drinking water (Fig. 3) [22].

Nitrate reaches the salivary glands through the bloodstream after being absorbed into the human body through the gastrointestinal tract (small intestine). In saliva, nitrates are reduced to nitrites by commensal bacteria, and then to biologically active nitric oxides [23]. The content of nitrates in saliva is about 10 times higher than nitrates in plasma [11]. Although commensal bacteria can also promote the formation of nitrosamines by catalysing the nitrosation of amines. In particular, this process can be enhanced by bacterial overgrowth in diseases in which hydrochloric acid secretion is disturbed [24]. Nitrates and nitrites are produced in the L-arginine/NO-synthase pathway.

L-arginine is metabolized to citrulline with the formation of NO in a pathway that is catalyzed by nitric oxide synthases [25]. In turn, after getting nitrates and nitrites into the stomach, they are metabolized in enzymatic systems such as: aldehyde oxidase or mitochondrial enzymes and non-enzymatic ones to NO and other biologically active compounds NO2 or N2O3 [7]. NO is also involved in the production of S-nitrosothiols, such as S-nitrosocysteine (Cys-NO) and S-nitrosoglutathione (GS-NO), which exhibit high biological activity. Similar to NO itself, they influence vasodilation and are involved in cellular and intercellular signalling, including vascular regulation and transnitrosation reactions [26].

### Nitrates in Food

The widespread use of nitrogen fertilizers, the oxidation of ammonia from hu-man and animal waste, and the treatment of water with chloramines have made nitrates a major contaminant in drinking water [7]. According to the standards in force in Poland, the permissible concentration of nitrates in water should not exceed 50 mg per liter [27]. The acceptable daily intake (ADI) of nitrates in Poland and the European Union is 3.65 mg/kg of body weight [28]. Nitrates and nitrites are also used as additives to food products such as processed meats or cured meats. They can also occur naturally in fruits and vegetables, with their concentration largely depending on seasonality and the cultivation system. For this reason, organically grown vegetables may contain lower concentrations of nitrates [7].

Research shows that as a result of vegetable consumption, the largest amount (about 80%) of nitrates is supplied in the diet. The nitrate content in the soil can vary geographically and seasonally. For example, the nitrate content in spinach can be twice as high in autumn and winter and in spring. The highest accumulation of nitrates is observed in the stems, peduncles, and leaves of plants [11]. Data analysis by Karwowska and Kononiuk from 2020 showed that arugula, spinach, and lettuce have the highest nitrate content. Beetroot and celery rank in the next place. Storage, heat treatment, and pickling may reduce the content of nitrates in vegetables and fruits [7].

The addition of nitrites to food contributes to the improvement of the taste and durability of the product by inhibiting rancidity and the development of *Clotridium botulinium*. Nitrates also improve the color of meat (pink color), which is why these compounds are often used in meat curing [11]. As a result of the addition of nitrite as a preservative, nitrosamines are formed in the reaction of amines with nitrites, which have a negative impact on the function of the cardiovascular and metabolic systems and are considered carcinogenic compounds [24]. For curing, sodium nitrite and/or potassium nitrate are most often used [29]. According to Rivera et al., there are no other substances that would have a similar effect in the product. Therefore, it is very difficult to eliminate nitrates from a product without compromising its quality. However, the current awareness of consumers has increased in recent years and they do not want to choose products where synthetic nitrates and nitrites have been used. For this reason, it is believed that the “uncured” label should be used correctly when the product actually qualifies for such a label and does not contain additives of these substances, even if substances of plant origin were used, e.g. from powdered celery [30, 31]. In 2019, the Centre for Science and Public Interest and Consumer Reports petitioned the USDA’s Food Safety and Inspection Service (FSIS) for labelling requirements of FSIS for meat and poultry products such as bacon, hot dogs, and pepperoni that have been processed with nitrates or nitrites [31]. However, the changes have not yet been implemented, and FSIS has announced that it will propose other changes and clarifications to pro-vide clear information to consumers. In addition, according to the North American Meat Institute, these compounds are still considered safe and play a significant role in protecting public health due to the prevention of botulism [32]. The uncured raw material contains a relatively small amount of nitrates and nitrites (approximately 2.5 mg/kg (variations of 0.15 to 11.12 mg NaNO2)/kg) and despite the addition of these compounds in subsequent processing, their permissible content is not exceeded. How-ever, the situation is different when the initial product contains a large amount of nitrates or nitrites (approximately 10 mg/kg) [11].

### Ingestion of Nitrates with Water and Food

Ward et al. in their review showed that there is still a lack of well-designed studies on the individual health effects associated with an increased risk of nitrate intake from drinking water [33]. According to van Den Bran et al., since nitrates are converted into nitrites in the human body, the risk of exposure should be assessed together with water and food. In all scenarios, total dietary exposure to nitrites exceeded the ADI of 0.07 mg nitrites/kg bw/day (mainly from vegetables, fruits, and fruit products). In addition, the combined dietary exposure to nitrates and nitrites exceeded the ADI for nitrites for each scenario, on average 1.7 times for all age groups and 3.5 times for children aged 1 year [34].

The study by Eliasz et al. showed that the average Intake of nitrites by Estonian children aged 2–35 months and 3–10 years was, respectively, 0.015 and 0.016 mg kg-1 bw day-1 with drinking water and meat products. Acceptable Daily Intake (ADI) 0.07 mg of nitrites kg-1 mc. day-1 was exceeded in 3.1% of children, more often in the young-er age group. Nevertheless, over the last 10 years in Estonia, the intake of nitrites in children’s diets from processed meat products has decreased [35]. A Polish study evaluating the national consumption of nitrates and nitrites in 2006–2012, based on data from the Central Statistical Office, showed that the average household consumption of nitrates and nitrites was 147 mg NaNO3 and 3.26 mg NaNO2/person/day, respectively, which corresponds to 41% and 45% of the ADI (acceptable daily intake), thus con-firming that the consumption of these compounds is at a safe level in Poland [36]. Gaj-da-Wyrębek et al. confirmed that the level of nitrates in radish, beetroot and cabbage is not a threat, however, they point out that high consumption of beetroot by children may result in exceeding the ADI [37]. Similar conclusions were obtained in a Belgian study among respondents aged 15 and over. Probably, higher consumption of vegetables and tap water (for brewing coffee, tea) may be associated with higher intake of nitrates [38]. In an Italian study by Roil et al., the highest concentrations of nitrates were found in rocket, radish, spinach, chard, lettuce, zucchini, and red chicory (> 40 mg/kg). Most of the tested samples did not exceed the permissible limit of Regulation EC No. 1333/2008. The same goes for “Bresaola” cold cuts and bacon. However, taking into ac-count the daily consumption of the tested products, relatively high consumption by children and infants was noted, exceeding the safe levels established by the JECFA 2002 Joint Food and Agriculture Organization of the United Nations/World Health Organization Expert Committee on Food Additives (2002) [39]. People following a vegetarian diet are also at risk for high intake of these compounds. Data from the study by Vlachou et al. showed that the estimated exposure to nitrites resulting from nitrate conversion was 210% of the ADI for people using such a diet. Nevertheless, there is great individual variability in nitrate conversion capacity [40]. In addition, it is likely that the presence of vitamin C in the stomach reduces the conversion of nitrite to N-nitroso compounds through the reduction of nitrite to nitric oxide, thus may prevent the adverse effects of nitrite [41].

The results of available studies indicate that the addition of nitrites and nitrites as food additives is often within safe levels for all population groups. However, special attention should be paid to the consumption of nitrates with vegetables and fruits by vegetarians and processed meat products such as cold cuts or sausages by infants and small children. Although in the case of the former mentioned group, the benefits of consuming vegetables and fruits may outweigh the risks associated with exposure to nitrates.

## Effect of Nitrates on Health and Physical Performance

### Sports and Exercise Performance

Athletes often use dietary supplements to improve their exercise capacity. Nitrates, through nitric oxide, can exert an effect on increasing performance and strength during exercise. Beet juice (BJ) has been classified by the International Olympic Committee alongside caffeine, β-alanine, creatine as a dietary supplement that can improve athletic performance when taken in the right dosage and in a specific type of exercise. Beet juice is a source of dietary fiber, minerals such as calcium, iron, sodium and potassium as well as a number of antioxidant compounds, including betalain. However, the effects of BJ on physical performance have been attributed to the high levels of inorganic nitrate found in this product (< 250 mg per 100 g of fresh BJ) [42, 43]. Maximum serum NO2 concentrations are seen 2–3 h after NO3 supplementation [44]. As mentioned earlier, NO3 can be converted to NO2 by bacteria in the oral cavity and by certain enzymes in the tissue. NO has been shown to effectively improve athletic performance, cause vasodilation, and improve blood flow at rest and during training. NO3 supplementation in the form of BJ increases blood flow to type 2 fibers leading to improvements in resistance training, modulates contractile function by modulating Ca2 + handling, increase mitochondrial function reducing oxygen cost during muscle contraction [43].

In a double-blinded, counterbalanced, crossover study design, acute supplementation with beetroot juice (BJ) at a dose of 70 ml (400 mg per dose) positively affected power, speed and number of repetitions during a dumbbell bench press with a free weight. Studies show that beetroot juice can be a safe and effective ergogenic agent to improve performance and optimize training results [45]. Similar results were observed by Ranchal-Sanchez et al. BJ supplementation resulted in an increase in repetitions on the day of administration of the preparation compared to placebo, although no differences were found for maximum speed and maximum power after BJ consumption and placebo. In both cases, BJ supplementation occurred 120 min before exercise. It is likely that such a procedure can provide significant benefits on muscle endurance during resistance training [45, 46] without changes in blood lactate levels in participants and in perceived fatigue [46], which is also confirmed by the earlier study by Mosher et al. This study showed that during high-intensity exercise, as a result of BJ supplementation, exercise tolerance is prolonged and phosphocreatine stores are used more effectively [47]. Studies also indicate that supplementation in a single dose or over several days can improve performance at intermittent, high-intensity efforts with short rest periods, and muscle power [48]. Therefore, it is suggested that nitrate supplementation may be used in a healthy male population to increase exercise tolerance and improve performance. However, long-term studies are still needed.

### Diseases of the Circulatory System

In studies, the consumption of beetroot juice, arugula drink and spinach had a positive effect on the concentration of nitrates and nitrites in plasma, thus reducing blood pressure [45, 49]. These products contain a number of other compounds, vita-mins, minerals, or flavonoids, which, through synergistic action with NO3, can im-prove endothelial function and affect the reduction of blood pressure. However, cur-rent data obtained from blood and saliva sample analysis indicate a significant in-crease in plasma concentrations of nitrate/nitrite after BJ consumption, which are indirectly associated with an increase in NO. In turn, increased NO production influences endothelial muscle relaxation through increased NO synthase activity [50]. In addition to improving endothelial function, consumption of inorganic nitrates may inhibit platelet aggregation, arterial stiffness, and protect against ischemia-reperfusion injury [51, 52]. Endothelial dysfunction is believed to be the initial stage of cardiovascular disease (CVD) development and may result from impaired production and/or availability of NO [52]. Flow-dependent vascular dilation (FMD) is directly related to endothelial function, and its increase is associated with reduced CVD risk. A meta-analysis by Bahrami et al. in 2021 confirmed that inorganic nitrate intervention improves both FMD and pulse wave velocity [53]. Similar observations were presented by Lara et al. However, in the case of elderly people with obesity and elevated blood pressure, the effect of inorganic nitrates on the improvement of endothelial function was smaller [54]. The positive effect of nitrates is mainly due to the formation of NO in the nitrate-nitrite-NO pathway. Nonetheless, there is still a lack of data from studies that would determine effective doses in hypertensive patients with cardiometabolic disease and whether nitrogen supplementation can be used in combination with antihypertensive therapy. In addition to the use of a diet rich in flavonoids [51], the supply of nitrates may have a beneficial effect on reducing the risk of CVD [51, 52] also in patients with chronic obstructive pulmonary disease. NO3 supplementation in this group of patients also had a positive effect on exercise performance during respiratory rehabilitation [55].

According to a 2018 meta-analysis of data by Jackson et al., the intake of inorganic nitrates may contribute to the reduction of CVD risk factors. In addition, it is believed that a reduction of resting blood pressure by 5 mmHg and diastolic blood pressure by 2 mmHg can reduce the risk of death due to coronary heart disease, stroke, and death in general [56]. However, long-term studies are still lacking, especially in the elderly, who are at high risk of developing CVD. Therefore, increased nitrate intake with the diet seems reasonable. A large amount of nitrate, as much as 174–1222 mg, is pro-vided by the Dietary Approaches to Stop Hypertension (DASH) diet [57], which is 550% of the daily allowable intake of nitrates for an adult weighing 60 kg recommend-ed by WHO [58]. However, according to the European Food Safety Authority (EFSA), a low intake of vegetables and fruits carries a greater risk than a higher intake of nitrates and nitrites [59]. In addition, the DASH diet has a fully confirmed positive effect on reducing the risk of CVD factors, therefore it is suggested that the presence of nitrates may contribute to such an effect [60].

Furthermore, optimising pathways that regulate NO production and S-nitrosothiol formation has been shown to be a promising therapeutic strategy for preventing and treating CVD. Nitrosothiols may support cardiovascular function by maintaining an appropriate redox balance in the heart, modulating vascular tone, and regulating blood flow [61]. However, more, large-scale clinical trials confirming the safety of strategies based on modulation of S-nitrosylation and a better understanding of the molecular mechanisms involved in the formation and action of S-nitrothiol are still needed to develop personalised therapies for CVD patients.

### Immune System and Intestinal Microbiota

In a study by Raubenheimer et al. after consuming beet juice with high nitrate content, CD11B expression on granulocytes was observed to decrease 3 h after consuming the juice. A statistically significant time x trial interaction effect was noted for intermediate monocytes with an increase in the intermediate population 6 h after ingestion of nitrate-rich juice compared to the group that consumed juice with low nitrate content (12.9 mmol vs. 0.01–0.04 mmol nitrate per serving). The effect of time was observed in classic monocytes. In addition, acute anti-adhesive and anti-coagulant reactions were noted, with a simultaneous decrease in blood pressure. The results may be useful in reducing the risk of developing aging-promoting vasculitis [62]. The use of nitrates may be important in regulating inflammation in a wide variety of pathophysiological conditions, including ischemia/reperfusion, vascular and gastro-intestinal injury, and toxemia [63].

A significant relationship is observed between inflammation and the intestinal microbiome [57]. The microbiota can be modified by many factors, including nutrients and non-nutrients supplied with food [64, 65]. Commensal bacteria can metabolize nitrates to produce compounds with a beneficial effect, which is NO. It is likely that the simultaneous administration of ascorbic acid together with nitrate may increase NO formation due to its ability to reduce ascorbic acid by reversing the reaction of nitrosating and nitrating agents. Therefore, it may be beneficial to combine foods containing vitamin C with products that are sources of nitrates [24]. Ascorbic acid is naturally found at high levels in fruits and vegetables, which makes nitrates and nitrites in these foods far less likely to form carcinogenic nitrosamines. In contrast, processed meats and dairy products contain much lower levels of ascorbic acid, leading to higher levels of nitrosamines. One practical way to mitigate nitrosamine formation in nitrate/nitrite-rich foods low in ascorbic acid is by adding lime or lemon juice, which are natural sources of vitamin C. Therefore, combining foods rich in vitamin C with nitrate-containing products may both increase the beneficial effects of nitrates and reduce carcinogenic risks.

In addition, the supply of probiotics and prebiotics may also contribute to increased NO signaling. Thus, the effect of various nutrients on the modulation of inflammation is observed. Chronic inflammation accompanies many diseases, such as obesity, metabolic syndrome, neuro-degenerative disorders, and type II diabetes [66]. In patients with uncomplicated diabetes, lower plasma nitrate levels are observed, although not in patients with diabetes and coronary artery disease, as well as microalbuminuria and normoalbuminuria or metabolic syndrome [67–70]. Studies by Henstridge et al. involving humans have shown that the supply of nitrates may have a positive effect on improving glucose up-take, regardless of the concentration of insulin in the plasma [71]. Recent research also focuses on the existence of the microbiota-muscle axis. Changes in the gut microbiota may also translate into exercise capacity, and exercise may modulate the composition of the gut microbiota and increase the production of short-chain fatty acids (SCFA). Skeletal muscle may be a reservoir of nitrates that are used after intense exercise, but the mechanisms responsible for this are still unknown. In turn, the resulting SCFA may participate in exercise adaptation [72].

Current reports indicate that the nitrate-nitrite-nitric oxide pathway may also affect intestinal inflammatory pathways through host-microbial redox signaling, which is important in inflammatory bowel diseases. NO may reduce, among others, the ex-pression of leukocytes, platelets, and endothelial cell adhesion molecules, including intercellular adhesion molecule-1 (ICAM-1). It is believed that nitrites can influence the composition of microbes in the intestines, thus ensuring that they can exhibit antimicrobial activity, including against *Helicobacter Pylori*, bacteria such as *Yersinia enterocolitica*, and *Salmonella enteritidis*. Probably, nitrate supplementation may increase the production of bactericidal molecules for local inflammation [65].

In summary, the use of nitrates can significantly reduce inflammation and reduce the negative effects of oxidative stress. The control of nutrient intake and their direct relationship to NO formation may be of great importance for the prevention of chronic inflammatory diseases.

## The Relationship of Nitrates with the Thyroid Gland

### Effect of Nitrates on Thyroid Diseases

The influence of nitrates on thyroid function has been of interest to scientists in recent years [73]. A 2022 systematic review found an increased risk of thyroid cancer with high dietary nitrate intake [74]. Probably, excessive consumption of nitrites from animal sources, especially processed meat (canned meat, bacon, etc.) by women, doubles the risk of developing thyroid cancer [75]. Additionally, this risk can increase in patients with thyroid dysfunction [76]. Studies evaluating the eating habits of patients with hypothyroidism confirmed higher consumption of processed meat than control groups [77, 78].

After the Chernobyl explosion, there are significant amounts of nitrates in the groundwater in the soil. There is an increase in the incidence of thyroid cancer among population groups living in those areas. In combination with radiation, children are at increased risk of thyroid cancer when exposed intrauterine or early childhood. This can lead to hypoxia and overproduction of NO [22]. Similar effects were seen in Wisconsin, United States. Based on test results from 2010 to 2017, 28% of the state’s residents who used private wells were exposed to nitrates. In this region, 111–298 cases of colorectal cancer and cancers of the thyroid, ovaries, kidneys, and bladder were observed annually. In the case of children, low birth weight in newborns, cases of premature birth and neural tube defects have been reported [79]. The results of a meta-analysis by Temkin et al. showed similar effects related to exposure to nitrates [80]. The Academy of Pediatrics recommends that pregnant and lactating women avoid exposure to excess nitrates, which may be present in contaminated well water and thiocyanide pre-sent in cigarette smoke [81]. A number of data confirm the relationship between the consumption of water contaminated with nitrates and the development of thyroid diseases [22, 33, 82], both metabolic and hormonal, as well as genotoxic damage in women [77]. There is probably an increased reduction of nitrates in the oral cavity in women, also after ingestion of inorganic nitrates [83]. However, according to the position of the American Association of Clinical Endocrinologists and American College of Endocrinology from 2015, there is no strong evidence to support the relationship between the development of cancer, autoimmune thyroid disease and the presence of nitrates in the diet [84].

### The Disturbing Effect of Nitrates on the Thyroid Gland

There are several potential mechanisms by which nitrates can disrupt thyroid function. First, nitrates can interfere with gastrointestinal iodine absorption by inhibiting Na+/K + ATP-ase [85]. The thyroid gland can concentrate monovalent anions, i.e. nitrates, leading to inhibition of iodine uptake [86]. Nitrates can also interfere with thyroid function by binding to the sodium iodide symporter, thereby interfering with iodide uptake by the thyroid gland. As a consequence, there is a increase in the levels of thyroid hormones (HT) and decrease thyroid stimulating hormone (TSH) through a negative feedback loop [84]. In a study of nursing mothers and newborns, exposure to nitrates has been shown to reduce iodine uptake and may result in increased TSH levels in newborns. This supports the assumption that maternal exposure to sodium-iodide symporter (NIS) inhibitors may have a negative impact on offspring thyroid function [87]. Combined exposure to perchlorates, nitrates, and thiocyanates may affect maternal thyroid function and TSH rise during pregnancy [88]. In addition, there is a possibility that exposure to these compounds may impair growth in girls aged 6–8 years. Nevertheless, further research on their relationship to childhood development is needed [89].

Some authors indicate a relationship between exposure to nitrates from drinking water and the development of subclinical hypothyroidism (SH). Analysis by Bivolarska et al. showed statistically significant negative correlation between iodine and thiocyanate levels in urine, positive correlation between nitrates and TSH and negative correlation between nitrates and FT4. The authors concluded that environmental pollution, e.g. nitrates have an effect on relative iodine deficiency, which can directly suppress the synthesis of thyroid hormones. These compounds can exacerbate iodine deficiency disorders in humans, especially in endemic regions of iodine deficiency. It has also been observed that a deficiency of selenium in combination with an increased amount of nitrates may have a negative impact on the iron and iodine status of postpartum women. In addition, high levels of nitrates pose a risk of developing methemoglobinemia, thus leading to the development of hemolytic anemia and premature birth or cyanosis [90]. According to the WHO report of 2003, if the demand for iodine in the di-et is covered at the recommended level (corresponding to the daily excretion of iodine of 150–300 µg/day), the effect of nitrates is weak, tending to zero. The effect of nitrates on thyroid function can be strong in the presence of dietary iodine deficiency [91]. Although the relationship between the concentration of nitrates in the urine and the thy-roid parameters has not been clearly confirmed [92].

Genetic factors may also be important in the development of thyroid disorders. FOXE1 as a transcription factor regulates the expression of thyroglobulin and thyroid peroxidase, thus leading to the synthesis of HT. Studies indicate that polymorphism in the FOXE1 gene may be associated with the risk of papillary thyroid carcinoma, follicular nodules and the occurrence of Bamforth-Lazarus syndrome [93]. Gandarilla-Esparza et al. analysed metabolic and hormonal alterations in women chronically exposed to nitrates in drinking water. The authors concluded that the presence of the A allele was associated with decreased TSH concentrations. The same effect was also observed for the FOXE1 rs1867277 polymorphism, suggesting that thyroid alterations may be influenced by the environment (nitrates in drinking water) and genetic factors (polymorphisms in the FOXE1 gene) and confirming the existence of gene-environment interactions. Thus, chronic exposure to nitrates from drinking water can be associated with the risk of developing subclinical hypothyroidism [82]. However, there is still a lack of data on the molecular mechanisms involved in the impairment of thyroid function by nitrates [94].

Numerous studies have shown that SH may pose a significant risk of cardiovascular disease, which occurs as a result of the development of inflammation and changes in the lipid profile leading to endothelial dysfunction [95]. The development of oxidative stress plays a major role in this process [96]. Reduced levels of nitrate and nitrite are observed in the course of CVD. Nitric ox-ide can be a reliable biomarker of the risk of cardiovascular disorders in the course of SH and be an indicator of the introduction and dosage of levothyroxine in replacement therapy [90]. Higher levels of nitric oxide are observed in thyroiditis and thyroid cancer, so in this case NO levels may also be a diagnostic biomarker for these disorders [97].

## Conclusions

While dietary nitrates can have beneficial effects by promoting cardiovascular health, improving exercise performance, and reducing blood pressure, excessive consumption of nitrites, especially from processed meats, has been linked to an increased risk of certain diseases.

In order to prevent chronic diseases and cancer, products such as sausages, ham or bacon should be consumed in limited amounts or even eliminated from the diet. It is particularly important to control the consumption of these products by infants and children, as the safe allowable intake of nitrates in their diet may very easily be exceeded. Conducting extensive training among parents and people involved in nutrition planning in nurseries and kindergartens to limit highly processed products containing nitrates is extremely important for the good health and proper development of children.

These contrasting effects highlight the importance of a balanced diet rich in natural sources of nitrates, such as vegetables, while minimizing the intake of processed meats to maintain overall health and reduce the risk of associated diseases in each age group.

Furthermore this approach to nutrition may also have a positive impact on ongoing climate change, since the consumption of animal products is associated with a larger carbon and water footprint. Therefore, it is important to properly educate the public and encourage people to eat more plant-based diets, such as the planetary diet proposed by The EAT-Lancet Commission.

## Data Availability

No datasets were generated or analysed during the current study.
